# Metastatic Small Cell Carcinoma Presenting as Acute Pancreatitis

**DOI:** 10.7759/cureus.8975

**Published:** 2020-07-02

**Authors:** Cameron Burmeister, Tamer S Said Ahmed, Mohammad Taleb

**Affiliations:** 1 Internal Medicine, University of Toledo, Toledo, USA; 2 Pulmonary/Critical Care Medicine, University of Toledo Medical Center, Toledo, USA

**Keywords:** small cell lung cancer, acute pancreatitis, mediastinal lymphadenopathy

## Abstract

Small cell carcinoma is a malignant lung cancer with poor prognosis that occurs almost exclusively in heavy smokers. Small cell cancer typically arises from the central airways, with the most common presentation being a large hilar mass with bulky mediastinal adenopathy. Small cell lung cancer rarely metastasizes to pancreatic tissue and presents as acute pancreatitis. Here, we describe a case of metastatic small cell lung carcinoma initially presenting as acute pancreatitis. The patient underwent CT of the abdomen, magnetic resonance cholangiopancreatography, and endoscopic ultrasound with biopsy which confirmed the diagnosis of small cell lung carcinoma. After positron emission tomography staging, the patient was subsequently treated with radiotherapy in tandem with multiple cycles of cisplatin and etoposide with positive treatment response.

## Introduction

Small cell lung carcinoma (SCLC) is a malignant lung cancer with poor prognosis known for its tendency to metastasize to both regional and distant organs aggressively. Acute pancreatitis is characterized by a combination of clinical symptoms, such as abdominal pain, elevation in pancreatic enzymes, and radiologic evidence of pancreatic inflammation or damage. SCLC commonly metastasizes to lymph nodes, lungs, brain, bone, and adrenal glands. However, SCLC can also rarely metastasize to the pancreas and cause acute pancreatitis [1]. Metastasis-induced acute pancreatitis (MIAP) is an extremely rare etiology for acute pancreatitis. Here, we present a case of MIAP secondary to incidentally discovered SCLC in a 73-year-old Caucasian male patient.

## Case presentation

**Figure 1:**
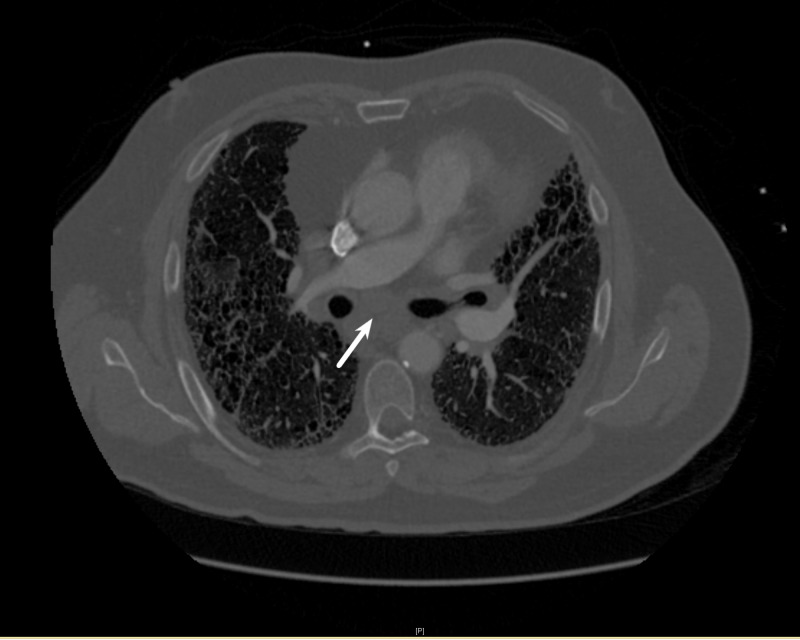
CT angiography of the chest showing subcarinal mass (arrow)

A 73-year-old man with a past medical history, including hypertension, diabetes mellitus type II, Global Initiative for Chronic Obstructive Lung Disease stage D chronic obstructive pulmonary disease, chronic respiratory failure on 2 L/minute of oxygen through a nasal cannula, and former tobacco user with >30 packs/year smoking history, presented with a one-week history of sharp, periumbilical/epigastric abdominal pain (8/10 on the visual analog pain scale) without radiation to the back. Associated symptoms included nausea without vomiting, anorexia, and fatigue; symptoms were exacerbated by movement and relieved with rest. The patient denied gallbladder disease, alcohol use, hyperlipidemia, trauma, history of pancreatitis, fever, recent illness, history of malignancy, occupational exposures, dietary changes, or recent travel. His family history was positive for small cell lung cancer in his mother as well as cancer in his sister and grandmother. This patient’s physical exam was notable for epigastric tenderness without hepatosplenomegaly, rebound tenderness, rigidity, jaundice, or right upper quadrant tenderness. His vital signs were within reference limits (heart rate 60 beats/minute, temperature 97.8°F, blood pressure of 117/50 mmHg, respiratory rate of 16 breaths/minute, and weight 102 kg). The patient’s laboratory results showed a white blood cell level of 13,300 cells/mcL, a hemoglobin level of 13.9 g/dL, hematocrit of 41%, a platelet count of 278,000 per mcL, a lactic acid level of 1.2 mg/dL, glucose of 169 mg/dL, blood urea nitrogen of 25 mg/dL, a creatinine level of 1.3 mg/dL, a sodium level of 134 mEq/L, a potassium level of 4.3 mEq/L, a chloride level of 103 mEq/L, a bicarbonate level of 20 mEq/L, a calcium level of 9.8 mg/dL, partial thromboplastin time of 32 seconds, an international normalized ratio of 1.0, a brain natriuretic peptide level of 25 pg/mL, a lipase level of 96 U/L, a triglyceride level of 150 mg/dL, a total bilirubin level of 0.4 mg/dL, an aspartate aminotransferase level of 14 U/L, an alanine aminotransferase level of 12 U/L, an alkaline phosphatase level of 45 IU/L, an albumin level of 4.1 g/dL, a troponin level of 0.01 ng/mL, and a procalcitonin level of 0.1 ng/mL. A CT of the abdomen/pelvis with contrast revealed fat stranding around the pancreatic head suspicious for acute pancreatitis; a gallbladder ultrasound showed no stones or sludge in the gallbladder but dilation of the pancreatic duct to 6 mm. A CT angiography (CTA) of the chest showed right paratracheal/hilar lymphadenopathy, as well as a 4.8 x 2.7 cm subcarinal mass (Figure 1). Our patient presented with acute pancreatitis complicated by mediastinal lymphadenopathy and an abdominal mass.

**Figure 2:**
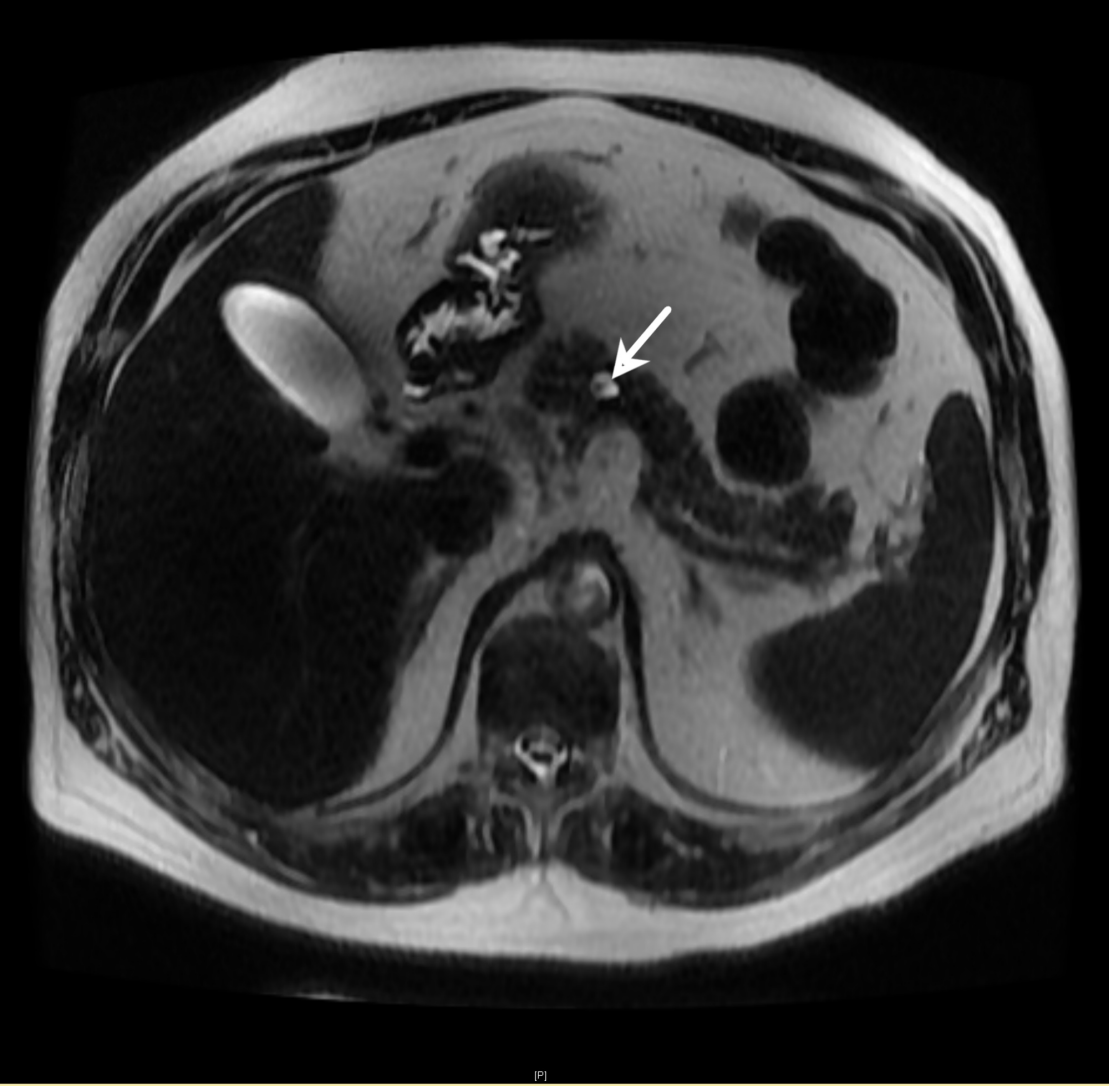
Magnetic resonance cholangiopancreatography showing lesion in the pancreatic body (arrow)

Magnetic resonance cholangiopancreatography (MRCP) showed two 1-cm lesions in the pancreatic body (Figure 2), lymphadenopathy, and inflammatory changes in the pancreatic head/uncinate process. The patient was discharged after abdominal pain and anorexia resolved, and he completed endoscopic ultrasound (EUS) with biopsy as an outpatient. The EUS confirmed mediastinal lymphadenopathy and showed that the two pancreatic lesions previously visualized on MRCP (8.1 x 7.6 mm and 6.1 x 4.3 mm) were communicating with the pancreatic duct. Fine needle aspiration (FNA) of the largest mediastinal lymph node (station 7) was sent for biopsy. The biopsy confirmed the diagnosis of metastatic small cell carcinoma.

**Figure 3:**
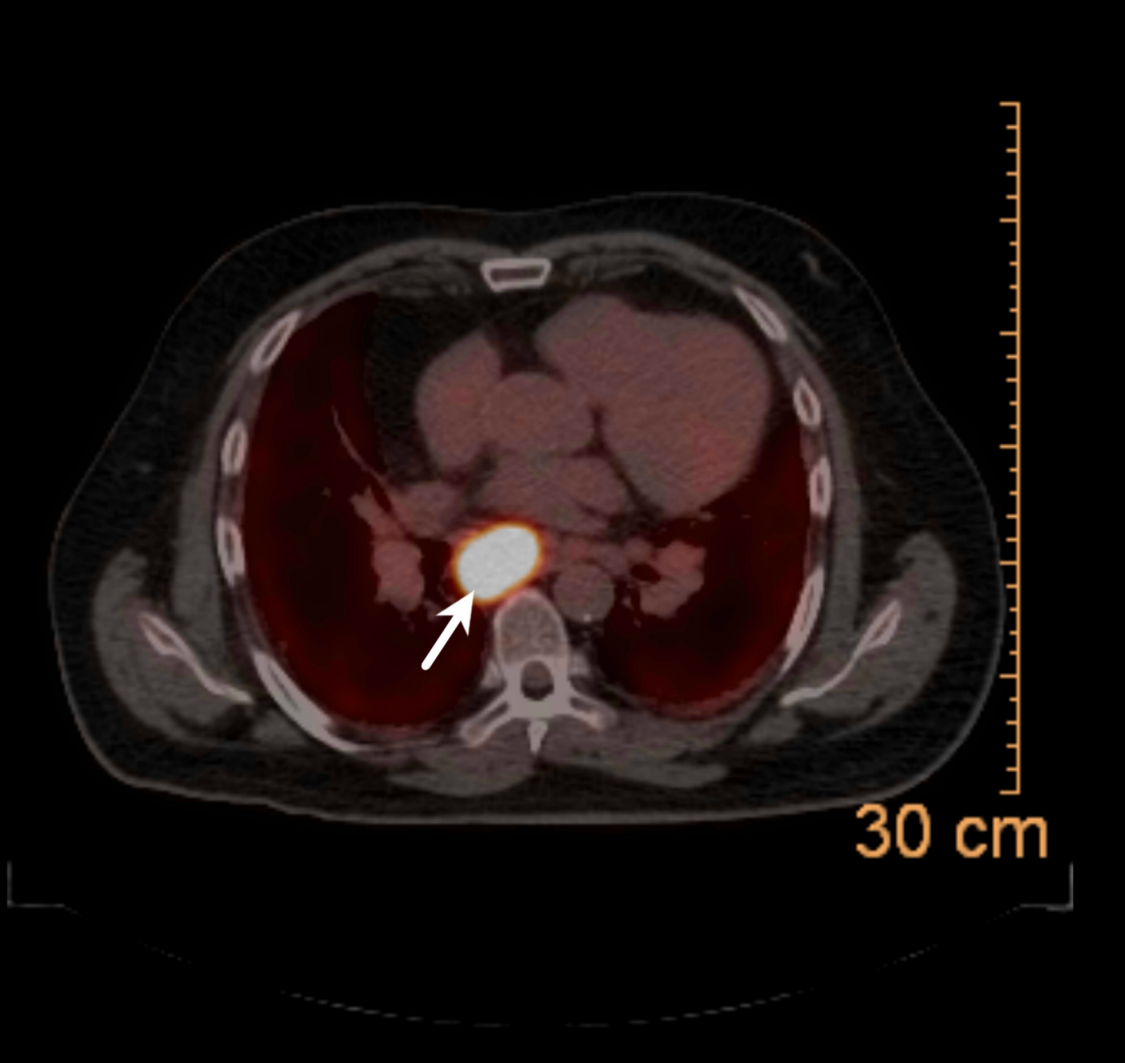
Positron emission tomography-CT showing avid lymph node in the subcarinal portion of the mediastinum (arrow)

The patient underwent an oncologic evaluation to determine the degree of metastasis of SCLC, and positron emission tomography (PET)-CT revealed an avid lymph node in the subcarinal portion of the mediastinum with moderate size lymph nodes throughout the mediastinum showing minimal PET activity (Figure 3).

The patient was started on cisplatin and etoposide every three weeks for up to six cycles with the first two cycles concurrent with radiation by oncology, and he tolerated his first two cycles with no significant complications.

## Discussion

Approximately 3% to 10.6% of all malignant tumors affect the pancreas [1]. The lung is the most common primary site for metastatic pancreatic tumors [2]. SCLC is the most common lung malignancy to invade pancreatic tissue, and postmortem autopsies of SCLC patients show metastases to the pancreas in up to 40% of cases [1-5]. Metastatic SCLC presents in three significant forms in the pancreas: as a well-circumcised lesion (50%-73%), generalized pancreatic enlargement (15%-44%), and as multiple small nodules (5%-10%) [5]. The pancreatic head and pancreatic body are the two most common sites of SCLC metastasis [6].

MIAP occurs via two major mechanisms: invasion/compression of the pancreatic duct from an intraparenchymal mass and/or extraparenchymal compression of the pancreas via adjacent lymphadenopathy. First reported in 1973 by Levine and Danovitch, MIAP is an infrequent complication of SCLC [5,7-10]. Retrospective studies of patients with lung carcinoma who developed MIAP note an incidence as low as 0.12%; however, the incidence of MIAP in patients with SCLC ranges between 0.4% and 7.5% of all SCLC cases in other retrospective analyses [3,5,9,11,12]. The most common symptoms of MIAP secondary to SCLC are abdominal pain and jaundice, likely secondary to pancreatic duct obstruction; however, 53% to 80% of patients with pancreatic metastases are asymptomatic at the time of incidental diagnosis by abdominal CT [5,6]. Our patient presented with abdominal pain, and the etiology of his MIAP was likely an extraparenchymal compression from mediastinal lymphadenopathy noted on chest CTA. While pancreatic metastasis often indicates advanced SCLC, MIAP rarely can be the initial presenting symptom of SCLC, as in this case [5]. EUS-FNA is sometimes employed for accurate evaluation and diagnosis of metastatic pancreatic lesions, and the diagnostic accuracy from FNA of metastatic pancreatic lesions is 89% to 92%; biopsy from FNA confirmed our patient’s diagnosis of SCLC [1,5,13]. Endoscopic retrograde cholangiopancreatography (ERCP) with pancreatic duct stenting has a role in improving both clinical symptoms and serum pancreatic enzyme levels for compressing metastases [1,5,13].

Unfortunately, SCLC carries a poor prognosis, and in a study of 20 patients with lung cancer and MIAP, the mean survival from MIAP diagnosis to death was 108.7 days. However, chemotherapy use and an Eastern Cooperative Oncology Group (ECOG) score < 2 had a clinically and statistically significant positive impact on survival time when compared to no chemotherapy and an ECOG > 2, respectively [14]. In SCLC patients, the median survival is approximately four to six months with chemotherapy, and as soon as two weeks from time of diagnosis with no treatment [5,14]. At the time of diagnosis, this patient had an ECOG score of 0 and was referred to oncology for chemotherapy, hopefully, to improve his overall survival.

## Conclusions

Acute pancreatitis is a common cause of abdominal pain in adult patients. Determining the etiology of acute pancreatitis is often difficult, as pancreatitis may develop via various dietary, environmental, autoimmune, anatomical, and even idiopathic factors. Accurate history-taking skills, with special attention to family history, social history, and associated symptoms, may be used in tandem with diagnostic laboratory and imaging data to include metastatic malignancy as a potential cause of acute pancreatitis. Metastasis-induced acute pancreatitis secondary to SCLC is a rare but significant disease process with a poor prognosis; however, accurate diagnosis and treatment of acute symptoms may improve clinical outcomes and possibly positively impact survival in affected patients.
